# Automated Video Analysis of Non-verbal Communication in a Medical Setting

**DOI:** 10.3389/fpsyg.2016.01130

**Published:** 2016-08-23

**Authors:** Yuval Hart, Efrat Czerniak, Orit Karnieli-Miller, Avraham E. Mayo, Amitai Ziv, Anat Biegon, Atay Citron, Uri Alon

**Affiliations:** ^1^The Theater Lab, Weizmann Institute of ScienceRehovot, Israel; ^2^The Department of Neuroscience, Sackler Faculty of Medicine, Tel Aviv UniversityTel Aviv, Israel; ^3^The Psychiatry Department, Chaim Sheba Medical CenterRamat-Gan, Israel; ^4^Department of Medical Education, Sackler Faculty of Medicine, Tel Aviv UniversityTel Aviv, Israel; ^5^Israel Center for Medical Simulation, Chaim Sheba Medical CenterRamat-Gan, Israel; ^6^Department of Neurology, Stony Brook University, New YorkNew York, NY, USA; ^7^Department of Theater, Haifa UniversityHaifa, Israel

**Keywords:** video analysis, doctor-patient interactions, performance, non-verbal communication, synchronization, entrainment

## Abstract

Non-verbal communication plays a significant role in establishing good rapport between physicians and patients and may influence aspects of patient health outcomes. It is therefore important to analyze non-verbal communication in medical settings. Current approaches to measure non-verbal interactions in medicine employ coding by human raters. Such tools are labor intensive and hence limit the scale of possible studies. Here, we present an automated video analysis tool for non-verbal interactions in a medical setting. We test the tool using videos of subjects that interact with an actor portraying a doctor. The actor interviews the subjects performing one of two scripted scenarios of interviewing the subjects: in one scenario the actor showed minimal engagement with the subject. The second scenario included active listening by the doctor and attentiveness to the subject. We analyze the cross correlation in total kinetic energy of the two people in the dyad, and also characterize the frequency spectrum of their motion. We find large differences in interpersonal motion synchrony and entrainment between the two performance scenarios. The active listening scenario shows more synchrony and more symmetric followership than the other scenario. Moreover, the active listening scenario shows more high-frequency motion termed jitter that has been recently suggested to be a marker of followership. The present approach may be useful for analyzing physician-patient interactions in terms of synchrony and dominance in a range of medical settings.

## Introduction

The quality of the physician-patient interaction is influenced by the affective-relational component of their communication. Studies show that this component can affect some aspects of patients' health outcomes (e.g. blood pressure and blood sugar levels, Kaplan et al., 1989) and the patient's evaluation of the physician (Ben-Sira, 1982; Griffith et al., 2003; Robinson, 2006).

The affective-relational dimension is primarily conveyed by non-verbal signals. Physicians' non-verbal behavior was shown to affect several aspects of patients' behavior, including self-disclosure, satisfaction, understanding of visit medical details, and adherence to medical recommendations (Larsen and Smith, 1981; Smith et al., 1981; Harrigan et al., 1985; Bensing et al., 1995; Hall et al., 1995; Duggan and Parrott, 2001; Robinson, 2006; Martin and DiMatteo, 2013). For example, physician gaze direction toward the patients increases self-disclosure by patients (Bensing et al., 1995; Duggan and Parrott, 2001). Patient satisfaction and understanding correlate with physicians orienting their body toward patients (Larsen and Smith, 1981; Smith et al., 1981). Patient's compliance increases with eye-contact, touch, close proximity, and leaning forward of physicians (Aruguete and Roberts, 2002).

Non-verbal communication is thought to enable good rapport through two main dimensions: affiliation and control (Kiesler and Auerbach, 2003). Affiliation is communicated by physician's warmth, caring, trust, and cooperation signals. It is established through eye-contact, smiling, nodding, close and frontal body positioning, synchronous motion etc. (Manusov, 2004). The dimension of control is conveyed by dominating, high-status behavior which is communicated through postural rigidity, visual dominance (gaze directed when speaking to as opposed to when listening to the other interlocutor), facial expressions (such as absence of smiling), standing in close proximity to the other, interruptions and long speaking times (Hall et al., 2005). Studies suggest that the higher the physician's affiliation and the lower the dominance, the better the patient's health outcomes (Stewart, 1995; Kiesler and Auerbach, 2003; Schmid Mast et al., 2008; Kelley et al., 2009; Martin and DiMatteo, 2013), although individual patients may vary in their preferences for doctor styles (Cousin and Schmid Mast, 2013).

It is therefore important to provide tools to measure and interpret non-verbal characteristics of physician-patient communication. At present, such tools rely mainly on human coding of videos of the interaction (Roter and Larson, 2002; Gallagher et al., 2005; Krupat et al., 2006; D'Agostino and Bylund, 2011). Widely used semi-automated software allows annotations of the interactions throughout the interaction (Caris-Verhallen et al., 1999; Ford et al., 2000; Roter and Larson, 2002). This software is used to study and debrief physician-patient interactions (Ziv et al., 2006, 2013). Both manual and semi-automated approaches require human coding which is labor intensive and hence limits the types of studies which can be carried out. An automated tool for the medical context would therefore be of interest.

Here, we show that an automated tool for measuring and analyzing non-verbal communication can be effective in a medical setting. Our tool brings to the medical field approaches that have been developed for automated analysis of general human interactions. This approach began in the late 1960's with detection of interactional synchrony in films of conversing people (Condon and Ogston, 1967; Kendon, 1970). In recent years, non-verbal signals recorded by video or depth cameras have been analyzed by computer vision tools for both synchrony and dominance effects (Feldman, 2007; Hung et al., 2007; Oullier et al., 2008; Gatica-Perez, 2009; Knapp and Hall, 2009; Alexiadis et al., 2011; D'Ausilio et al., 2012; Delaherche et al., 2012; Cristani et al., 2013; Won et al., 2014; Volpe et al., 2016). Synchrony is usually measured between the velocities or energies of motion of the two communicators. Synchronous motion has been shown to correlate with positive affect and sense of connection between the conversants (Lakin et al., 2003; Baaren et al., 2004; Wiltermuth and Heath, 2009). Dominance can be assessed by the imbalance of turn-taking and the relative duration of speech turns (Delaherche et al., 2012). Recently, in a study on moments of togetherness in joint improvisation (Noy et al., 2011) an additional marker for followership was suggested: at moments of followership, follower motion is characterized by a “jittery” pattern, where the follower velocity weaves around the leader's velocity at relatively high frequencies, in the range of 1.5–5 Hz (Noy et al., 2011). This high-frequency motion is termed jitter (Hart et al., 2014; Noy et al., 2015).

We present automated analysis of non-verbal synchrony and dominance in a medical setting, as part of a larger experiment (Czerniak et al., 2016) designed to study the impact of doctor's performance on the placebo response (Kaptchuk et al., 2008; Kelley et al., 2009). We demonstrate video analysis markers that discern between two types of doctor behavior.

## Methods

### Scenarios of interaction

Healthy volunteers were recruited from the community, ostensibly to participate as subjects in the evaluation of a new analgesic ointment (hand moisturizer with no analgesic components). This study was done with the approval of the local institutional review board (IRB) as well as by Israel's Ministry of Health ethics committee. Subjects met a professional actor portraying a physician. The actor presented the “drug” and asked the subject to apply the ointment. The actor did so with a performance chosen at random from two scripted and rehearsed scenarios called scenario A and B (see Czerniak et al., 2016 for more details). We thus compared two performances: (A) “disengaged and detached” scenario: actor looks mainly at computer screen and types, asks a few closed questions. (B) “engaged and suggestive” scenario (Stewart, 1995; Matusitz and Spear, 2014): actor asks open questions, actively listens with an attentive body posture and reflects answers.

Each scenario was based on research on the performance of healing and effective physician-patient communication (Bensing and Verheul, 2010; Martin and DiMatteo, 2013). In addition to verbal text, the scenarios specify body language indications including posture dynamism, movement in space (physician's office), proximity to the subject, eye-contact with the subject, vocal volumes, tempo, and intonation. The scenarios are described in detail in the Appendix.

### Subjects

Forty-three subjects' videos were analyzed in the study, of which 34 were male and 9 female. Subjects' age ranged between 18 and 39 years, with mean of 24 ± 6 years. Education ranged between 12 and 18 years, with mean of 14 ± 2 years. Twenty-one subjects participated in scenario A and 22 subjects in scenario B. Subjects in both scenarios had similar age and education levels (scenario A: age:23 ± 6 years, education: 13 ± 2 years, scenario B: age: 24 ± 5 years, education: 14 ± 2 years). Scenario B had 7 female participants while scenario A had 2 female participants. However, analysis of male subjects alone (being the majority group in both scenarios) showed a similar significant difference between male synchronization and mutual followership values in scenario B compared with scenario A (Mann–Whitney test, *p* < 0.002, see **Figures 2, 3** for whole group analysis results). For more details on subject demographics see (Czerniak et al., 2016).

### Videos of actor-subject interactions

**Figure 1:**
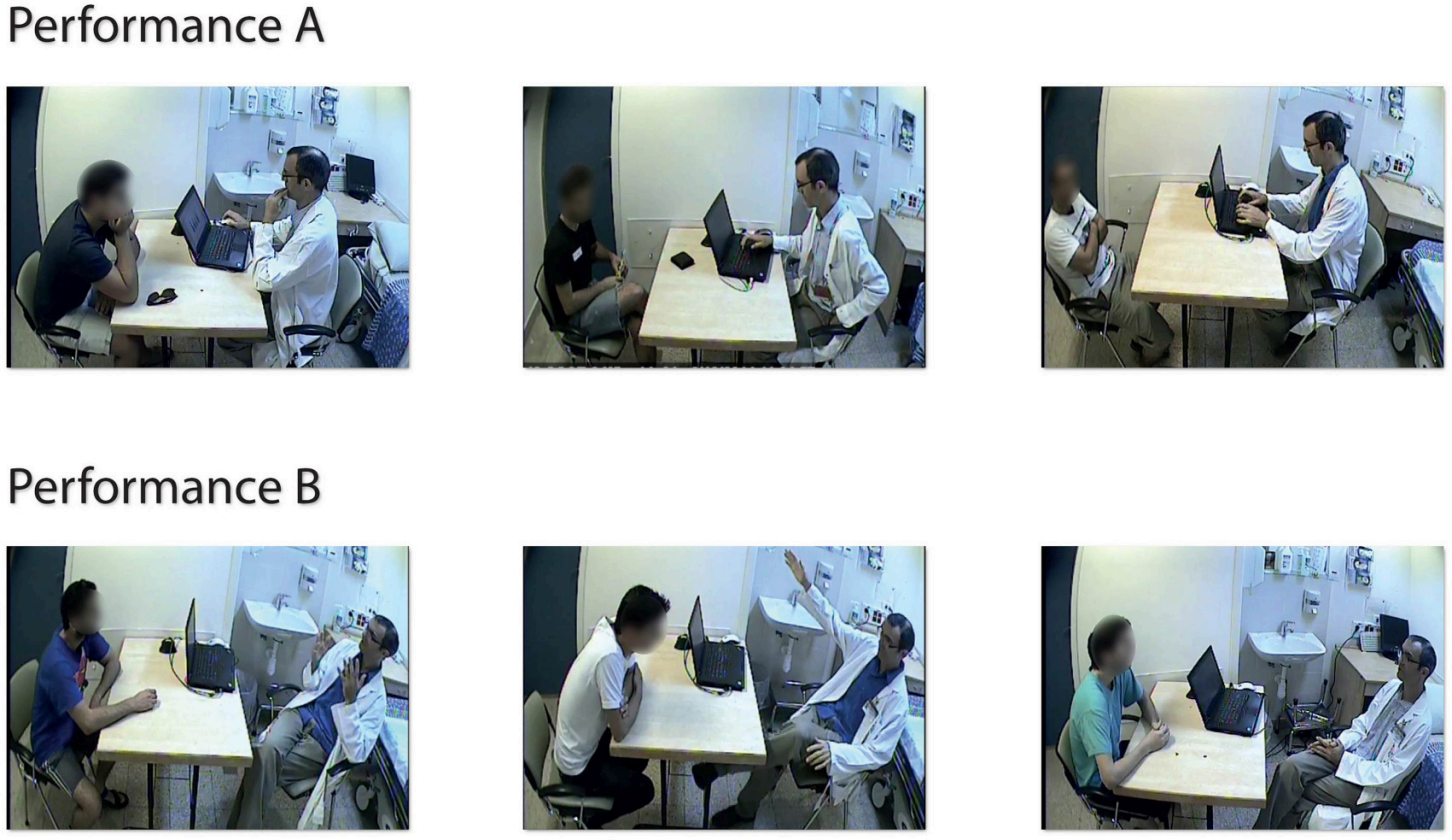
**Examples of performance A and B in the dyadic actor-subject interaction**. In performance A, the actor mainly types, and asks a few closed questions. In performance B, the actor actively listens to the subject using open questions and reflections, and explains the mechanism and effect of the drug.

We analyzed movies of actor-subject interactions sitting facing each other with a desk between them in a typical medical office setting (Figure 1). This data is part of a larger study (Czerniak et al., 2016), in which different camera positioning were used to film actor-subject interactions. Preliminary analysis showed that the 43 videos with a camera position 1 m to the side and at a height of 1.7 m (Figure 1) was optimal for video analysis. The other videos were filmed at an angle in which one of the participants was partially occluded. Each of these 43 videos was analyzed from the moment when both the subject and actor sit in their chairs, up to the moment before the actor reaches for the analgesic ointment. The duration of the analyzed interactions ranged between 123 and 379 s (210 ± 49 s, mean ± std).

### Automated video image analysis tool for non-verbal communication

We computed the velocity of each pixel in each frame, namely its movement from one frame to the next, using an optical flow algorithm (Black and Anandan, 1993). Each movie was divided down the middle of the desk into a subject part and actor part of the frame. The total energy of the pixels of each part of the frame (sum of squared pixel velocities) was attributed to the subject and actor accordingly.

We analyzed the cross-correlation between the subject and actor energies. The cross-correlation function is:

$$\begin{array}{l} \text{c}\left(\tau \right)=\overset{N-\tau -1}{\underset{n\;=\;0}{\sum }}E_{s}\left(n+\tau \right)E_{A}\left(n\right)/std\left(E_{S}\right)std\left(E_{A}\right) \end{array}$$

where *E*_*s*_(*n*) is the subject's kinetic energy and *E*_*A*_(*n*) is the actor's kinetic energy at frame n.

From c(τ) we calculated (i) motion synchrony (Feldman, 2007; Delaherche et al., 2012), the kinetic energy cross-correlation at zero lag, c(0), and (ii) total and instantaneous entrainment and leading/following behavior, equal to the cross correlation function center of mass, $\frac{\int_{-T}^{T}tc\left(t\right)dt}{\int_{-T}^{T}c\left(t\right)dt}$, where the cross correlation is calculated over the entire movie or over a moving window of 20 s.

In addition, we calculated the power spectrum of the motion using the Fourier transform of the energy, which describes what portion of the kinetic energy comes from the motion at each frequency. To measure jitter, motion suggested to characterize followership (Noy et al., 2011), we analyzed the total power at high frequency (1.5 Hz and above).

### Classifier for performance scenarios

We used a classifier based on the synchrony (denoted x) and mutual followership (denoted y), with a probability function *P* = 1/(1+*ae*^*bx*+*cy*^). Parameters were set by bootstrapping the dataset with replacements and fitting to a logistic regression classifier. The parameters of the logistic regression classifier are: *a* = 5 ± 1, *b* = −23 ± 5, *c* = 0.6 ± 0.2, mean ± std.

## Results

### Engaged doctor performance scenario shows more synchrony and more symmetric followership

This study considers physician behavior as a form of performance (Goffman, 1959; Schechner, 2012) which can be defined and manipulated. We trained an actor to portray a doctor with two possible scenarios: scenario A was disengaged and detached, and scenario B was engaged and suggestive (see Sections Methods and Appendix). We analyzed videos of encounters with 43 different subjects, 21 from scenario A and 22 from scenario B. We measured the kinetic energy of the actor and subject in each frame, and evaluated their synchrony and followership using cross-correlation of their motion (see Section Methods). The cross correlation function at lag τ measures the extent to which the energy of the subject at a given moment is correlated with the energy of the actor at a time τ in the past. Thus, it measures the similarity in activity at different lag times. At zero lag, the cross-correlation function indicates the immediate synchrony between the actor and the subject, denoted c(0). At positive lags, the cross-correlation indicates an entrainment of the subject to actor's motion, as occurs when the subject moves a few seconds after the actor. At negative lags, it indicates the reverse: followership of the actor after the motion of the subject.

**Figure 2:**
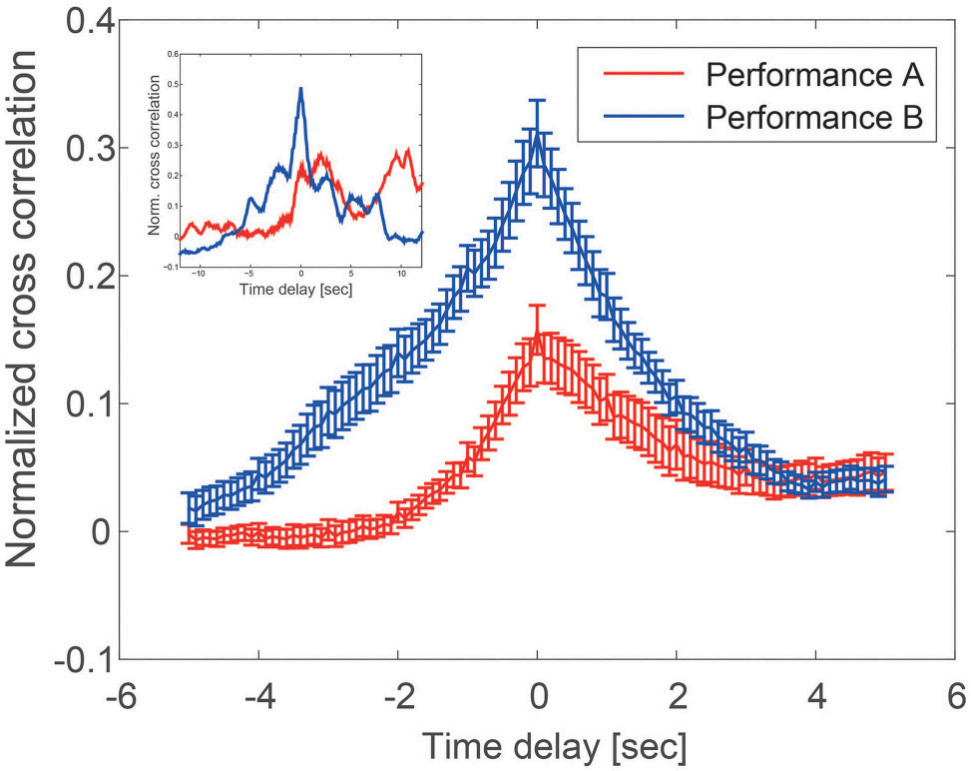
**Cross-correlation of subject and actor motion kinetic energy shows higher synchrony and symmetric followership in performance B (blue) compared with performance A (red)**. Height at zero delay means synchrony of motion, height at positive delay means subject's entrainment by the actor, and height at negative delay means actor's entrainment by the subject. x-axis: time delay [sec], y-axis: Normalized cross-correlation. Inset, two examples of specific dyadic cross-correlation of performance A (red) and performance B (blue).

The cross correlation function for scenario A and scenario B is shown in Figure 2. Motion synchrony is higher in scenario B than in scenario A [c(0) = 0.33 ± 0.03 vs. c(0) = 0.14 ± 0.02, mean ± ste, *p* < 0.001]. This can be seen in Figure 2, where the peak cross-correlation at zero lag is higher in scenario B than A.

We further find that scenario B showed a symmetric decay of cross-correlation at positive and negative lags (the symmetric tent-like shape of the blue curve in Figure 2). In contrast, scenario A showed a non-symmetric shape weighted on average toward positive lags. This indicates that in scenario B, actor and subject follow each other's motion in turns, whereas scenario A shows one-way followership: the subject tended to follow the actor in most videos (Figure 2).

**Figure 3:**
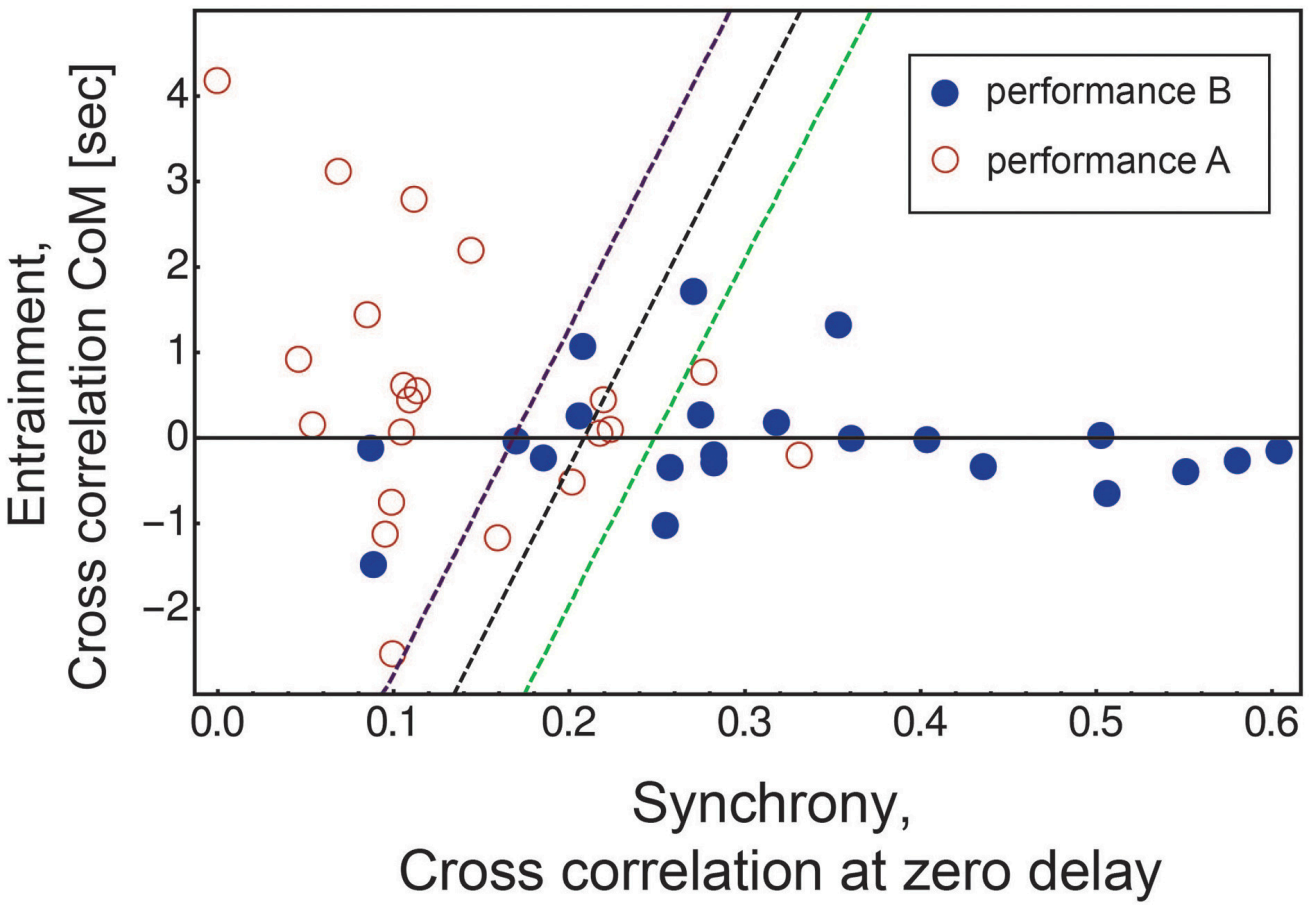
**Synchrony and entrainment of dyadic interaction differentiates between performance A and B in the video analysis**. Performance B (blue circles) has higher synchrony values and more equal entrainment between the actor and subject compared with performance A (red circles). A logistic regression classifier separates the two performances with a 72% accuracy (black dashed line). The 70% probability function lines for performance B (green dashed line) and for performance A (purple dashed line) are shown. The classifier probability function can be described as: *P*(1) = 1/(1 + *ae*^*bx*+*cy*^). The parameters of the logistic regression classifier are: *a* = 5 ± 1, *b* = −23 ± 5, *c* = 0.6 ± 0.2, mean ± std, as determined by bootstrapping with 1000 repeats.

To ask whether these two indicators—synchrony and mutual-followership—robustly differentiate the two scenarios, we constructed a logistic regression classifier based on synchrony and asymmetry (see Section Methods). The classifier correctly classified 72% ± 7% of the videos (mean ± std, bootstrap). The classifier can be visualized by the dashed lines in Figure 3.

**Figure 4:**
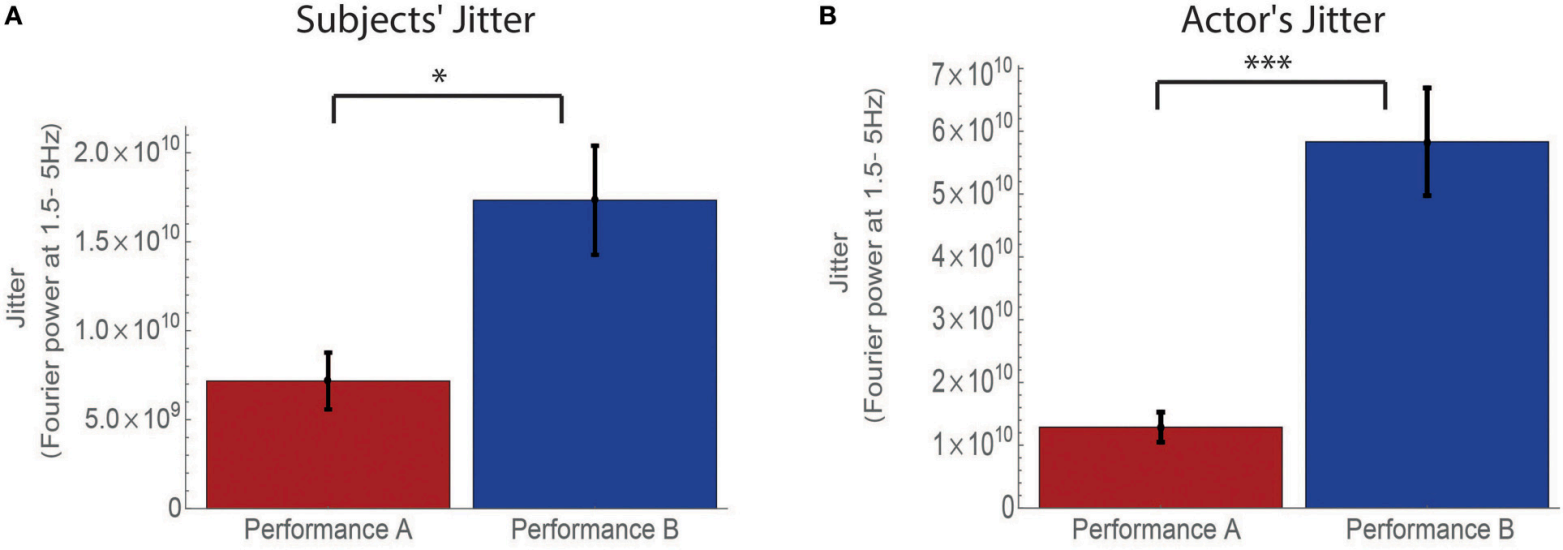
**Jitter of subjects and actor differs between performance A and performance B**. The jitter motion (Fourier Power at frequencies 1.5–5 Hz) of both subjects **(A)** and actor **(B)** is higher at performance B scenario, suggesting more followership motion (Noy et al., 2011) of both interlocutors. ^*^p < 0.05, ^***^p < 0.001. Subjects: Mann–Whitney test, *p* < 0.03, rank biserial correlation, *r* = 0.39. Actor: Mann–Whitney test, *p* < 0.001, rank biserial correlation, *r* = 0.8.

We also analyzed jitter as a marker of followership. We measured jitter as the motion at frequencies of 1.5 Hz and higher in the power spectrum of the subjects' kinetic energy. We find that subjects in performance B have more jitter than subjects in performance A (Mann–Whitney test *p* < 0.03, rank biserial correlation, *r* = 0.39, Figure 4A). This is also the case when analyzing the motion of the actor (Mann–Whitney test, *p* < 0.001, rank biserial correlation, *r* = 0.8, Figure 4B). This finding further supports the dual followership in scenario B observed in the cross-correlation signature.

We also tested turn-taking in the interactions. For this purpose, we calculated the cross-correlation function over a moving window of 20 s across the entire video. The center of mass of the cross-correlation function at each window indicates which person dominates this specific part of the interaction. We calculated the mean duration of bouts where the subject dominates the interaction and the mean duration of bouts where the actor dominates. We find that the subject-actor dominance ratio, defined as the ratio of mean duration of sequential dominance periods of either subject or doctor, is higher in performance B [performance A duration ratio = 0.98 ± 0.06 (mean ± ste), performance B duration ratio = 1.24 ± 0.09 (mean ± ste), Mann–Whitney test, *p* < 0.03, rank biserial correlation, *r* = 0.4]. This finding indicates that more equal turn-taking occurs in performance B compared to performance A.

## Discussion

We presented an automated method that can robustly provide time-resolved scores for non-verbal communication in a dyad within a medical setting (Ji and Liu, 2010) from standard video recording. Our method can detect the dyadic effects of the two interaction scenarios. It indicates higher synchrony and symmetric followership (lack of one-sided dominance) in performance B (“engaged and suggestive”) vs. performance A (“disengaged and detached”). Thus, the different performances induce different dyadic interaction which is recognized by our quantitative indicators.

The automated analysis method presented here does not require labor-intensive human coding nor specialized training. It also allows quantitative aspects such as motion frequency components to be captured. The large amount of data that can be analyzed allows good statistical validity. For example, the standard errors of synchronization in the present study are on the order of 10% whereas the effect size for synchrony between the two performances is larger than 1, yielding a *p*-value lower than 10^−3^. This compares well with human coding studies which produced inter-rater correlations ranging between 0.53 and 0.96 in the non-verbal, affective gesture categories with *p* < 0.01 (Caris-Verhallen et al., 1999; Nelson et al., 2010; D'Agostino and Bylund, 2011).

In this study, we used scripted performances of an actor for increased control of the interaction and as a way to obtain large differences between the types of doctor-patient interactions. Our automated method suggests that dyadic motion characteristics of synchrony and mutual-followership are key components differentiating between the two performances. It will be important to further examine the proposed analysis tool in a non-simulated medical setting, with multiple different doctors and patients across a range of natural occurring interaction types.

Future work can address further quantitative measures of physician-patient non-verbal communication. For example gaze orientation and body posture coupled with the analysis of momentary subject and doctor entrainment may allow a deeper understanding of the interaction. Additional experiments can address in a more fine way which aspects of the performance are possible active ingredients to enhance synchrony and turn taking. Performance includes both verbal text and body language components. One possible extension is to switch some aspects of the verbal component of scenarios A and B while maintaining the essence of their body language components. Other possibilities include separating different components of the performance such as active listening which builds rapport and authoritative explanation which builds suggestion.

More generally, automated analysis of physician-patient interaction can offer high-temporal resolution to debrief physicians and to study performance aspects of doctor-patient interactions. We hope that such research will guide training of clinicians in order to improve the way physicians interact with their patients toward better treatment outcomes.

## Author contributions

YH, UA, AB, AZ, and AC conceived the research, EC, OK-M, AZ, and AB gathered data, YH, AM, UA, EC, and AB analyzed the data, YH, AM, UA, EC, OK-M, AB, AC, and AZ participated in writing the paper.

### Conflict of interest statement

The authors declare that the research was conducted in the absence of any commercial or financial relationships that could be construed as a potential conflict of interest. The reviewer TC and handling Editor declared their shared affiliation, and the handling Editor states that the process nevertheless met the standards of a fair and objective review.

## Appendix: A detailed description of scenarios A and B

### Scenario A

The doctor busies himself typing on his laptop just before the volunteer knocks on the door. He says “come in” without looking at the person entering and without greeting that person unless the volunteer greets first, in which case the doctor responds with a greeting. He motions to the volunteer to sit down across the desk as he continues to type, his eyes still on the computer screen. Typing, which continues for another minute, is interrupted by the ringing of the doctor's cell phone. He takes it out of his pocket, studies the screen, and shuts it off, replacing it in his pocket. He continues to type for a few more seconds, then asks the volunteer for his/her name and finds it in his computer files. Now he looks at the volunteer for the first time and asks if he/she has just gone through the CPT. He asks to have a look at the hand that was immersed in the ice water, examines it visually from across the desk, asks to see the back of the hand and examines it briefly as well. He asks the volunteer what made him volunteer for the study and pretends to type the answer (payment/a friend's recommendation, etc.) on his laptop. He then rolls on his chair toward a chest of drawers, opens the top drawer and pulls out a small plastic jar containing moisturizer cream, stating that this new pain relief medicine is being tested and the volunteer should apply it evenly on both sides of the hand that has been in the ice water. He oversees the action performed and then asks the volunteer to proceed to the other room where the 2nd CPT round will take place.

### Scenario B

When there is a knock on the door, the doctor rises, walks toward the door, greets the volunteer by name, shakes his/her hand (except for orthodox women) and invites him/her to come in. He asks the volunteer to sit down across the desk and takes his seat on the other side. He asks what made the volunteer participate in the study and types the answer quickly on his laptop, resuming eye contact with the volunteer right away. He asks about the volunteer's experience during the CPT, listens to the answer and repeats it. He asks to examine the hand that was in the ice water, takes it in his own hand, and looks at it carefully while touching it on both sides. He then asks the volunteer to describe the pain he has just experienced, guiding him/her to use a metaphor or an image to communicate the particular feeling (e.g., like a knife cutting the flesh or like a burn). He continues by asking how the volunteer normally deals with pain. He listens to the answer and reflects it briefly, then proceeds to say that as a doctor, he has been studying pain and people's reactions to it for many years and has come to the conclusion that pain is a very personal experience that calls for a treatment that is designed individually for each person suffering from it. He says that this is the approach used in the present study, and that the new pain relief cream being tested is the product of many years of research in both Western and complementary medicine. He adds that the cream has different formulae, each designed for a different type of personality. He looks at the computer screen, as if studying the volunteer's answers to the questionnaire, and while rising from his seat, says that according to the answers he has just read, the type of cream that would work most efficiently in this specific case, would be …He pauses before completing the sentence, stands up turns to open the top drawer and carefully chooses one of the many plastic jars in it. He does this with his back turned to the seated volunteer. He then turns around, holding the jar above his head and hands it to the volunteer with a large gesture. He explains that the cream should be evenly applied on both sides of the hand, and adds that he is convinced that it is going to be very effective in reducing the pain during the second CPT. He then escorts the volunteer to the door.

For the full scripts see the Supplementary Material of Czerniak et al. (2016).
